# CHANGE IN USE OF REMOTE HEALTH TECHNOLOGIES AMONG OLDER AFRICAN AMERICAN MEN DURING THE COVID-19 PANDEMIC

**DOI:** 10.1093/geroni/igad104.2964

**Published:** 2023-12-21

**Authors:** Ledric Sherman, Janae Alexander-Bady

**Affiliations:** Texas A&M University School of Public Health, College Station, Texas, United States; Texas A&M University School of Public Health, College Station, Texas, United States

## Abstract

It is well known that minority communities are disproportionately affected by disparities present within the US health care system. With the onset of COVID-19, these gaps in health quality continue to be exacerbated. Understanding health technology use differences pre-COVID and post-COVID among African American men is a necessary first step towards improving health outcomes that may be targeted via health technology interventions. The purpose of this study was to explicate and contextualize post-COVID health technology use among African American men in the United States for self-management of health. Of the 54 participants, all between the ages of 65-85, who completed a 30-item technology use questionnaire, a majority reported having type 1 diabetes and high blood pressure and that their current health was good or very good. For technology devices used pre-COVID, the highest reported devices owned were smartphones, desktop computer, laptop, and fitness tracker. Post-COVID, participants purchased fitness tracker CGM, glucose meter, and scale to check weight. As researchers and providers work towards addressing the prevalent health disparities disproportionately affecting members of our communities, these results provide an informative description of pre- and post-COVID.

